# Screening of Potential Xanthine Oxidase Inhibitors in *Gnaphalium hypoleucum* DC. by Immobilized Metal Affinity Chromatography and Ultrafiltration-Ultra Performance Liquid Chromatography-Mass Spectrometry

**DOI:** 10.3390/molecules21091242

**Published:** 2016-09-17

**Authors:** Hong-Jian Zhang, Yi-Juan Hu, Pan Xu, Wei-Qing Liang, Jie Zhou, Pei-Gang Liu, Lin Cheng, Jin-Bao Pu

**Affiliations:** Key Laboratory of Research and Development of Chinese Medicine of Zhejiang Province, Zhejiang Academy of Traditional Chinese Medicine, 132 Tianmushan Road, Hangzhou 310007, China; jian871211@sina.com (H.-J.Z.); huyijuan604@163.com (Y.-J.H.); xpan840520@163.com (P.X.); jxlwq22@163.com (W.-Q.L.); Kenzei@163.com (J.Z.); lpgzaas@163.com (P.-G.L.); Lemon2193@163.com (L.C.)

**Keywords:** *Gnaphalium hypoleucum* DC., XO inhibitor, immobilized metal affinity chromatography, luteolin-4′-*O*-glucoside, luteolin

## Abstract

In this study, a new method based on immobilized metal affinity chromatography (IMAC) combined with ultrafiltration-ultra performance liquid chromatography-mass spectrometry (UF-UPLC-MS) was developed for discovering ligands for xanthine oxidase (XO) in *Gnaphalium hypoleucum* DC., a folk medicine used in China for the treatment of gout. By IMAC, the high flavonoid content of *G. hypoleucum* could be determined rapidly and efficiently. UF-UPLC-MS was used to select the bound xanthine oxidase ligands in the mixture and identify them. Finally, two flavonoids, luteolin-4′-*O*-glucoside and luteolin, were successfully screened and identified as the candidate XO inhibitors of *G. hypoleucum*. They were evaluated in vitro for XO inhibitory activity and their interaction mechanism was studied coupled with molecular simulations. The results were in favor of the hypothesis that the flavonoids of *G. hypoleucum* might be the active content for gout treatment by inhibiting XO.

## 1. Introduction

Gout is a common disease worldwide, mostly in developed countries [1]. The major treatments for gout include xanthine oxidase inhibitors and uricosuric drugs depending on the patient [2]. Xanthine oxidase (XO) is a critical enzyme that catalyses the oxidation of hypoxanthine to xanthine and further catalyzes the oxidation of xanthine to uric acid. The enzyme plays a vital role in the development of hyperuricemia and gout [3]. Allopurinol is a XO inhibitor clinically used in the treatment of gout. Although remarkably effective, its serious adverse reactions limit its usage [4]. As a result, the recent trend has been toward developing safer and natural XO inhibitors, as new inhibitors with higher therapeutic potency and less adverse effects are desired.

Chinese herbs have been used in the treatment of gout and hyperuricemia for a long time. *Gnaphalium hypoleucum* DC. is an annual ethnic medicinal herb that is widely distributed in the temperate regions of China. *G. hypoleucum* has been used as a folk medicine for its anti-inflammatory, antitussive, gout and expectorant activities, etc. [5]. Moreover, a previous study demonstrated that the extract of *Gnaphalium affine* D. Don from the same genus plant significantly reduce blood uric acid in rats [6], which inspired us to try to identify whether *G. hypoleucum* has the same efficacy and the active substances of that plant.

Phytochemical investigations of the plant revealed the presence of flavonoids as major constituents and that most of them are 5-OH flavanones [7,8]. Normally, researchers have used macroporous resins to enrich the total flavonoids of the genus *Gnaphalium*. However, the total flavonoid content was no more than 60% and the process was tedious and poorly efficient [9,10]. Meanwhile, reports suggested that flavonoids exhibited remarkable XO inhibitory activity [11]. We and mainly focused on flavonoids to investigate the new XO inhibitors from *G. hypoleucum*. Screening and identifying bioactive contents in complex mixtures is challenging, therefore, there is an urgent need to develop a simple, rapid, targeted and accurate method for screening of XO inhibitors from *G. hypoleucum*.

To our knowledge, 5-OH flavanones have a high tendency to chelate metal ions. Based on this theory, people have used metal salt and complexing agents to obtain total flavonoids [12,13], but the method was unrepeatable and unstable. In this research the objective was to obtain a product with high flavonoid content efficiently, simply and rapidly by immobilized metal affinity chromatography (IMAC) [14]. Moreover, ultrafiltration combined with liquid chromatography-mass spectrometry (UF-LC-MS) has been widely considered as a simple, rapid and low sample consumption method for the discovery of bioactive contents from complex mixtures [15,16]. Herein, we conducted immobilized metal affinity chromatography and ultrafiltration-ultra performance liquid chromatography-mass spectrometry (IMAC-UF-UPLC-MS) in vitro investigations to identify the XO inhibitors rapidly and efficiently, in order to clarify the potential pharmaceutical basis for the use of *G. hypoleucum* in treating gout and hyperuricemia.

## 2. Results

### 2.1. Binding and Elution of Flavonoids from IMAC Columns

**Figure 1:**
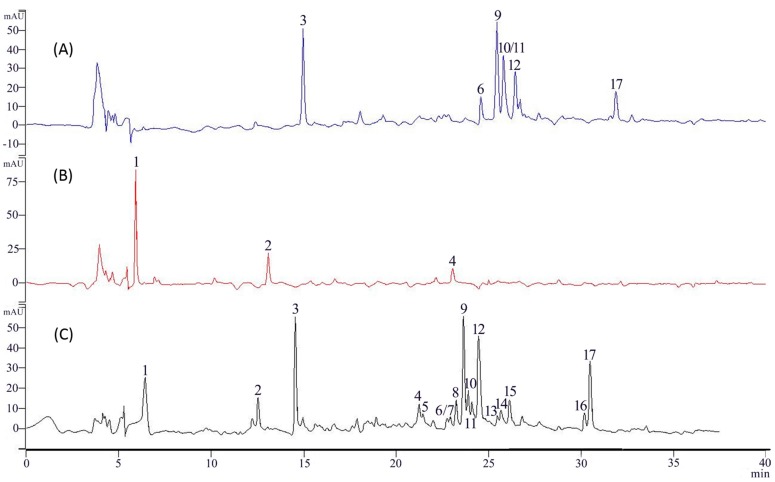
The HPLC chromatograms at 250 nm are as follows: (**A**) *G. hypoleucum* IMAC-Zn 1% HCl-MeOH eluent; (**B**) *G. hypoleucum* IMAC-Zn water eluent; (**C**) *G. hypoleucum* extract.

In IMAC column experiments, the sample solution was a mixture dissolved in 50% MeOH aqueous solution, washed sequentially with pure water and 1% HCl-MeOH. HPLC (Figure 1) shows the main contents were flavonoids, and a relatively high flavonoid content (1% HCl-MeOH eluate) and low flavonoid content (water eluate) were found, but the same sample loadings show different results using different metal ions (Supplementary Materials (SM)). 

Almost the same *G. hypoleucum* extract HPLC results were found after Mg^2+^/Al^3+^/Cr^2+^ and CTS-SiO_2_ without metal column chromatography of the water eluate (SM). In addition, the flavonoid content of the eluate from the Cu^2+^/Fe^3+^ column was lower than with Zn^2+^. The best complexation ion was Zn^2+^, and the extract absorption capability of CTS-Zn was 250 mg/g as detected by UV. Seventeen components were separated and detected at 250 nm in the extract of *G. hypoleucum* and seven major flavonoids of 1% HCl-MeOH eluent after CTS-Zn (Figure 1). The flavonoid content in the fractions collected from CTS-Zn was determined by UV. The recovery rate of flavonoid was 85.2% (SM). The concentration of the Zn^2+^ ions in the resulting solution was 17.725 µg/g (SM).

### 2.2. Validation of XO Inhibitory Activities

The *G. hypoleucum* extracts and 1% HCl-MeOH eluent of CTS-Zn column showed inhibitory effect on XO activity. The IC_50_ values of extracts and the standard drug allopurinol are displayed in Table 1.

### 2.3. Screening of XO Ligands by IMAC-UF-UPLC-MS

**Figure 2:**
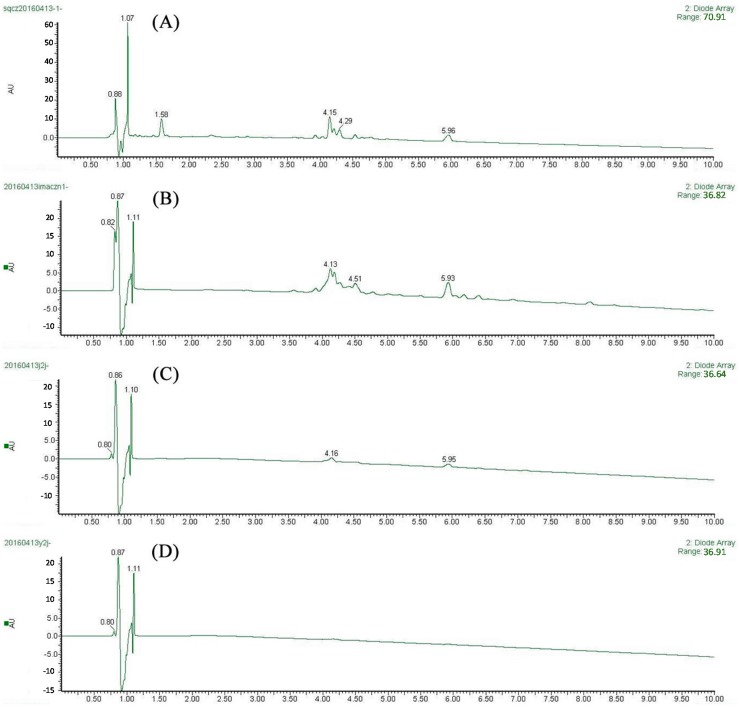
The UPLC chromatogram of *G. hypoleucum* extract (**A**) and IMAC 1% HCl-MeOH eluent (**B**) monitored at 250 nm; The UF-UPLC-MS method approach for screening selective ligands to XOD from IMAC-Zn 1% HCl-MeOH eluent (**C**) and *G. hypoleucum* extract (**D**).

**Figure 3:**
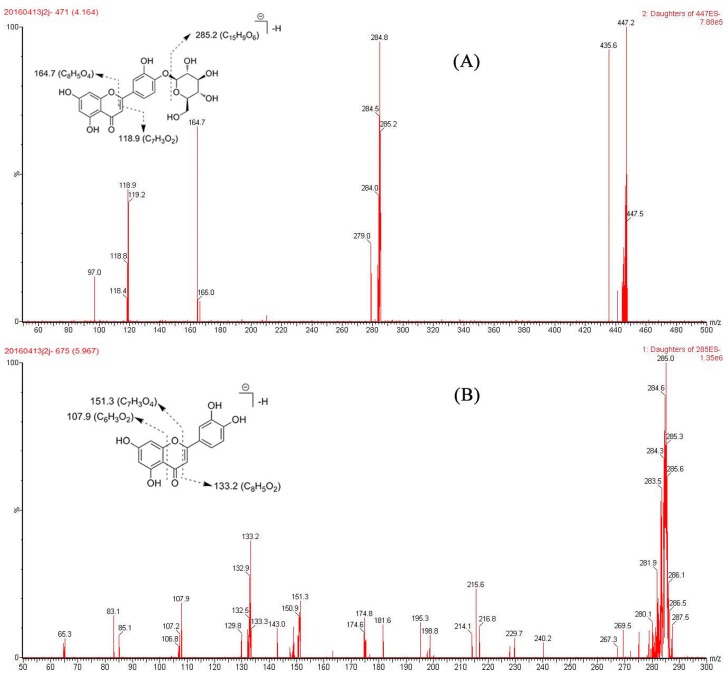
Chemical structure and fragmentation pathway of luteolin-4′-*O*-glucoside (**A**) and luteolin (**B**).

After ultrafiltration screening, two main peaks in the ultrafiltration chromatogram were distinguished (Figure 2C) but not identified in *G. hypoleucum* extract (Figure 2D) under the same conditions. Three independent experiments were performed, showing good repeatability. In Figure 2C, two faint peaks appeared at around 5 and 6 min which were detected by MS^2^ spectra (Figure 3). The molecular ions of the unknown compounds in negative mode were 447 and 285, which combined with DAD indicated that they were flavonoids.

### 2.4. Identification of Active Compounds

**Figure 4:**
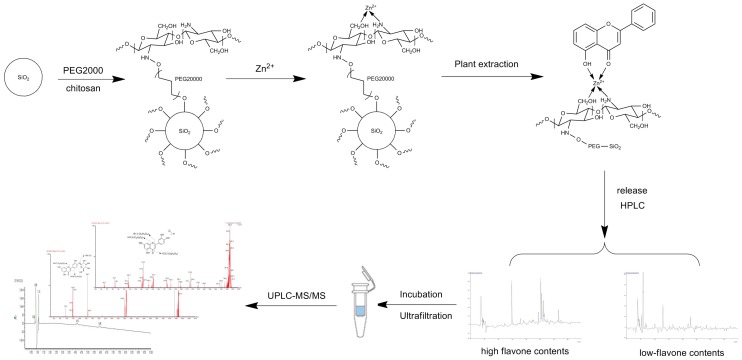
Schematic diagram of the IMAC-UF-UPLC-MS method.

After the candidate ligands were found, UPLC coupled with diode-array detection (DAD) and tandem quadrupole mass (TQ-MS) were applied for structural identification. By comparing the Rt, UV spectra and *m*/*z* of characteristic ions with the reference compounds and literature data [15,16], the two compounds were unambiguously identified as luteolin-4′-*O*-glucoside and luteolin by comparison of their retention times, UV and mass spectrometric data, summarized in Table 2, with those of the reference compounds (SM). The typical fragmentation ions of the flavonoid could be seen in these compounds (Figure 3). The IMAC-UF-UPLC-MS process is described in Figure 4.

### 2.5. Inhibition Active and Type of Screen Compounds on XO Activity

**Figure 5:**
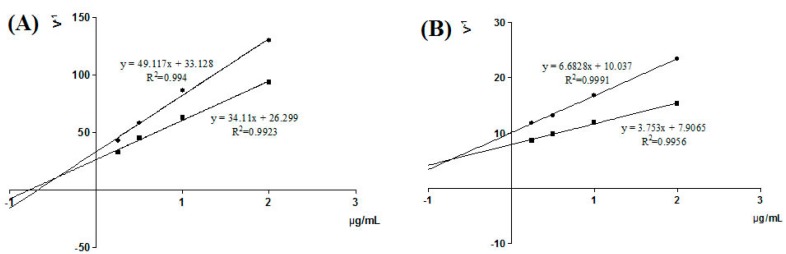
Inhibitory effect of luteolin-4′-*O*-glucoside (**A**) and luteolin (**B**) on xanthine oxidase. ● xanthine 100 µmol/L ■ xanthine 200 µmol/L.

Luteolin-4′-*O*-glucoside and luteolin had IC_50_ values of 0.26 µg/mL and 0.43 ug/mL, respectively. Luteolin-4′-*O*-glucoside exhibited greater XO inhibitory activity than luteolin. In addition, enzyme kinetics were analyzed by Lineweaver-Burk plots (Figure 5). From the intersection of the two lines, the K*i* values were calculated to be 0.455 μg/mL and 0.727 µg/mL for luteolin-4′-*O*-glucoside (Figure 5A) and luteolin (Figure 5B), indicating that these compounds were mixed inhibitors.

### 2.6. Computational Docking of Luteolin-4′-O-glucoside and Luteolin on XO

**Figure 6:**
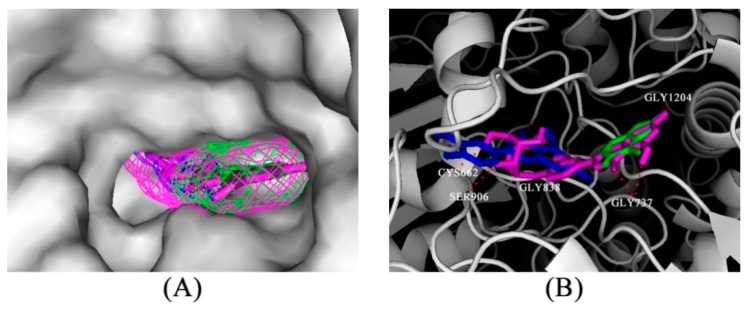
(**A**) Predicted binding mode of luteolin-4′-*O*-glucoside (pink), luteolin (blue), and allopurinol (green) docked into XO using the Autodock 4.2 software (Molecular Graphics Laboratory, La Jolla, CA, USA); (**B**) The interaction between luteolin-4′-*O*-glucoside (pink), luteolin (blue), allopurinol (green) and XO. The dashed lines (red) represent hydrogen-bonding interactions between luteolin-4′-*O*-glucoside (pink) and XO.

For predicting the binding mode between luteolin-4′-*O*-glucoside and luteolin with XO, a molecular docking study was conducted. From prior kinetic assays, we learned the screen compounds were mixed inhibitors; therefore, we focused on the active site of XO (Figure 6A) as in [17]. The docking results showed that the estimated binding energies of luteolin-4′-*O*-glucoside and luteolin were −8.89 and −7.32 kcal/mol and the estimated inhibition constants were 304.88 nM and 4.34 µM, respectively. This agrees with the experimental result that luteolin-4′-*O*-glucoside had better inhibitory activity than luteolin. Figure 6B shows that luteolin-4′-*O*-glucoside (pink) showed an interaction with GLY1204 and GLY737 like allopurinol. On the other hand, according to the structural information of XO (3ETR), there were some other interactions with residues GLY838, CYS662 and SER906. Obviously, once the cavity is plugged by inhibitors, this might block the attachment of substrate and ultimately prevent the catalytic ability of XO.

## 3. Discussion

A novel method coupling immobilized metal affinity chromatography (IMAC), ultrafiltration and ultrafiltration-ultra performance liquid chromatography-mass spectrometry (UF-UPLC-MS) was developed to discover XO inhibitors in *G. hypoleucum*. The IMAC technique can facilitate effective enrichment of the flavonoids from complex mixtures. Moreover, UF-UPLC-MS was used to distinguish the ligands from the high flavonoid content fraction. As a result, two flavonoids from *G. hypoleucum*—luteolin-4′-*O*-glucoside and luteolin—were successfully screened and identified by this method as XO inhibitors. Meanwhile, the in vitro inhibition of XO by all the fractions and identified compounds were investigated. It suggested that *G. hypoleucum* and its components possess XO inhibitory activity that might be helpful in preventing or slowing the progress of gout. The flavonoids may be the active components of *G. hypoleucum* in treating gout and hyperuricemia. The identified luteolin-4′-*O*-glucoside and luteolin exhibited significant inhibitory activity on XO, and they were mixed inhibitors. Furthermore, the identified luteolin-4′-*O*-glucoside and luteolin could be promising XO inhibitors for clinical use in the prevention and treatment of gout and hyperuricemia and to verify this further in vivo studies are being carried out.

## 4. Materials and Methods

### 4.1. Reagents and Materials

*Gnaphalium hypoleucum* DC. was collected from Yuhuan, Zhejiang Province, PR China, in October 2014. It was identified by the licensed pharmacist Yi-bo Feng, Tongde Hospital of Zhejiang Province and the voucher specimens (QSQC20141001) was deposited at the Key Laboratory of Research and Development of Chinese Medicine of Zhejiang Province, Zhejiang Academy of Traditional Chinese Medicine. Xanthine oxidase (X1875-5UN) from bovine milk, xanthine (X7375) and allopurinol were obtained from Sigma (St. Louis, MO, USA). DMSO, HPLC-grade acetonitrile, methanol and formic acid were purchased from Merck (Darmstadt, Germany). The standard compounds luteolin and luteolin-4′-*O*-glucoside (purity >98%) were isolated by our group and identified by NMR and MS. Water was purified through an Arium 611UV system (Sartorius Stedim Biotech, Göttingen, Germany). Solvents and all other chemicals were of analytical grade, polyethylene glycol with two molecular weights (PEG 20000) and chitosan (95% deacetylation) were purchased from Sinopharm Chemical Reagent Co., Ltd. (Shanghai, China). Silica gel (200–300 mesh size) was purchased by Qingdao Haiyang Chemical Co., Ltd. (Qingdao, China). The Power Wave XS 96-well UV plate reader was purchased from Bio-Tek (Winooski, VT, USA). An Amicon^®^ Ultra-0.5 10k filter column was obtained from Millipore Corp. (Bedford, MA, USA). Zn single element standard solution (GBW08620) was purchased from the National Standard Material Research Center (Beijing, China).

### 4.2. Apparatus

HPLC apparatus (Varian Inc., Santa Clara, CA, USA) with DAD detector. Acquity Ultra Performance LC-system (Waters, Milford, MA, USA) with TQ detector.). Vacuum oven (VACUCELL; MMM Group, Planegg, Germany). Eppendorf Centrifuge 5430R (Eppendorf, Hamburg, Germany). UV-vis spectrophotometer (Cary 100, Varian Inc.). Flame atomic adsorption spectrometer (240AA, Varian Inc.), equipped with a GTA 120 graphite tube atomizer and a PSD120 programmable sample dispenser.

### 4.3. Preparation of Extract of G. hypoleucum

The dried *G. hypoleucum* (100 g) was extracted twice using 500 mL MeOH (2 × 30 min) at room temperature under ultrasonication. Then solvents were removed with vacuum rotary evaporation to yield 13.3 g of residue. The residue was stored at 4 °C for further experiments. Before use, a portion of the residue was dissolved in phosphate buffer (50 mM, pH 7.5) combined with DMSO (<0.5%) to obtain a solution with a concentration of 0.2 mg/mL (referred to the raw material).

### 4.4. Preparation of IMAC Matrix (Metal-CTS-SiO_2_)

The method of coating of silica gel with cross-linking chitosan (CTS-SiO_2_) was used as previous report and the best coating solution was employed: 2% chitosan and 10% PEG 20000 [14]. After coating of CTS-SiO_2_, 10 g CTS-SiO_2_ were mixed with 200 mL of aqueous solutions containing 0.1 M Cu^2+^/Mg^2+^/Al^3+^/Zn^2+^/Cr^2+^/Fe^3+^ at constant pH of 5.0 (adjusted with HCl and NaOH), respectively. The flask was stirred magnetically at 100 rpm for 1 h (sufficient to reach equilibrium) at room temperature. The ion immobilized silica was filled in a column and washed with sufficient amount of pure water to remove the unbound metal ions. Finally, the IMAC column was obtained.

### 4.5. Preparation of High Flavonoid Content Fraction Measurements and Characterizations

The *G. hypoleucum* extract (1 g) was dissolved in 50% MeOH aqueous solution (20 mL) and loaded on a 10 g IMAC column. Then the column was washed sequentially with pure water and 1% HCl-MeOH (30 mL each), respectively, and the eluate collected for analysis by HPLC. The CTS-SiO_2_ without metal columns was used as control group. The HPLC separation was carried out using a 250 mm × 4.6 mm YMC 5 μm C_18_ column. The column was kept at room temperature. The mobile phase consisted of 0.1% formic acid aqueous solution (A) and acetonitrile (B) using a gradient elution of 15%–60% B at 40 min. The flow rate was set at 1.00 mL/min. The detection wavelength was 250 nm. Based on the HPLC analyses and UV detection of the flavonoids after using the IMAC column (SM), CTS-Zn showed the best preparation result. The maximal adsorption capacity of 0.2 g CTS-Zn was determined using a mixture with five different extract concentrations (12.5–100 mg/mL). Then UV was used to analyze the results of the adsorption capability test (SM). Moreover, the concentration of the Zn ions in CTS-Zn was determined by flame atomic adsorption spectrometry (240AA, Varian Inc.). The amount of adsorbed Zn ion was calculated by using the concentration of the metal ions at the equilibrium (SM).

### 4.6. Determination of XO Inhibitory Activity in Vitro

The enzymatic activity assay was carried out in a 96-well plate format according to the reported method with slight modifications [18]. First, 5 unit XO in buffer (40 µL, 0.5 U/mL sodium pyrophosphate/HCl, pH 7.5) and 3, 5, 10, 20, 40 µL of the test extracts in DMSO and phosphate buffer were incubated at 37 °C for 3 h. The reaction was started by adding 0.4 mmol/L xanthine in buffer (40 µL) to the mixture. The reaction mixture was incubated at room temperature and the absorbance at 293 nm was determined every 5, 10, 15, 20, 25, 30 and 60 min. Allopurinol was used as a positive control. The relative activity of XO was calculated as follows: inhibition (%) = [(rate of control reaction − rate of sample reaction)/rate of control reaction] × 100%. The assay was done in triplicate and IC_50_ values were calculated from the graph plotted as inhibition percentage against the concentration.

### 4.7. Screening Protocol for Selective Inhibitors of XO

Based on the XO inhibitory activity test results, the active extracts of *G. hypoleucum* and high flavonoid content fraction (1% HCl-MeOH eluent) (20 µL each) were incubated with XO (20 µL) for 30 min at 37 °C. After that, each mixture was filtered through a 10 kDa molecular weight cut-off membrane-based centrifugal filter device by centrifugation at 10,000 r/min for 10 min. The XO-ligand complexes were washed with 100 μL phosphate buffer solution by centrifugation three times at 10,000 r/min for 10 min. The washed XO-ligand complexes were dissociated using 200 μL 50% MeOH aqueous solution with a 15 min ultrasonication followed by centrifugation at 10,000 r/min for 10 min in a fresh ultrafiltration cell for three times. After centrifugation, the ultrafiltrate which contained the XO-ligands was used for UPLC-MS/MS analysis. All assays were performed in triplicate.

### 4.8. Screening Procedure of IMAC-UF-UPLC-MS

A 20 μL aliquot of the ultrafiltrate was analyzed on a ACQUITY™ Ultra Performance LC System (Waters, Milford, MA, USA). The separation was carried out using a 2.1 mm × 100 mm ACQUITY UPLC^®^ BEH 1.7 μm C18 column. The column was kept at room temperature. The mobile phase consisted of 0.1% formic acid aqueous solution (A) and acetonitrile (B) using a gradient elution of 15%–60% B at 10 min. The flow rate was set at 0.25 mL/min. The detection wavelength was 250 nm. LC-MS analysis was carried out using a Waters TQ Detector equipped with an electrospray ionization (ESI) interface. The ESI source was operated in negative mode, and the mass range was set at *m*/*z* 100 to 1000 with mass measurement of all mass peaks. The data were acquired and analyzed under Masslynx Workstation Software (version 4.1.896).

### 4.9. Identification of XO Ligands by UPLC-DAD-ESI-MS^2^

The multistage mass spectra (MS^2^) provided by ion trap mass spectrometry (IT-MS) could confirm the relationship between precursor and daughter ions. The structural of unknown compounds can be determined by this type of analysis. In this study, UPLC-DAD-ESI-MS^2^ was used to identify the ligands screened from ultrafiltration [15]. Prior to ESI-MS, parameters such as drying gas flow-rate, capillary tip voltage, and ion-source temperature were optimized for the ligands. The mass spectral data of the IMAC-UF fraction were obtained in negative ion mode. The compounds were identified by comparing the retention time (Rt), DAD and MS spectra with standard compounds and literature data [15,16].

### 4.10. Kinetic Analysis for Identified Inhibition

The same method was used to detect the inhibition activity of screened compounds [18]. The inhibition mode of the screened compounds was determined by a Lineweaver-Burk plot [17]. The concentrations of the inhibitor (0.25, 0.5, 1.0 and 2.0 µg/mL) were tested with two different concentrations of substrate (100 and 200 µmol/L). Each experiment was performed in triplicate.

### 4.11. Inhibitory Mechanism Study of XO Ligands

Molecular modelling studies were performed using the docking program AutoDock (vers. 4.2.6), to explore the probable binding interactions of screen compounds with XO. The X-ray crystal structure of XO (PDB code 3ETR) was used for the docking studies [19]. Docking simulations were performed using the Lamarckian genetic algorithm. From the docking results, the best scoring (i.e., with the lowest docking energy) docked model for a conformation was chosen [20]. To implement docking simulations, a grid box was defined to enclose the active site with dimensions of 40 Å × 40 Å × 40 Å and a grid spacing of 0.375 Å. The grid maps for energy scoring were calculated using AutoGrid. Default parameters were used except for the maximum number of docking runs (100). The output from AutoDock was rendered with PyMol (version 1.5.0.3.).

### 4.12. Statistical Analysis

The experimental results including XO activity assay and kinetic analysis for competitive-type inhibition were expressed as mean ± standard deviation (*n* = 3) and the data were analyzed using the GraphPad Prism software 5.0 (GraphPad Software, Inc., San Diego, CA, USA) and Microsoft Excel (Microsoft Office 2010, Microsoft, Redmond, WA, USA).

## Figures and Tables

**Table 1:** Inhibitory effect of samples and allopurinol on XOD activity in vitro.

Samples	IC_50_ (µg/mL)
*G. hypoleucum* crude extract	14.36
IMAC-Zn 1% HCl-MeOH eluent	3.43
IMAC-Zn Water eluent	23.58
Allopurinol	0.75
DMSO (Control)	-

**Table 2:** Retention times (Rt), UV, MS data for identification of XOD inhibitors in *G. hypoleucum* by UPLC-MS.

Rt (min)	UV	MS	Elem Comp	Fragmentation Pathways	Identification
4.16	367.1	447.2	C_21_H_19_O_11_	[M − H]^−^	
		284.8	C_15_H_9_O_6_	[M – H − Glc]^−^	Luteolin-4′-*O*-glucoside
		164.7	C_8_H_5_O_4_	[M – H – Glc − C_7_H_4_O_2_]^−^	
		118.9	C_7_H_4_O_2_	[M − H − Glc − C_8_H_5_O_4_]^−^	
5.95	347.1	285.2	C_15_H_9_O_6_	[M − H]^−^	
	253.1	151.3	C_7_H_3_O_4_	[M − H − C_8_H_6_O_2_]^−^	Luteolin
		133.2	C_8_H_5_O_2_	[M − H − C_7_H_4_O_4_]^−^	
		107.9	C_6_H_3_O_2_	[M – H − C_9_H_6_O_4_]^−^	

## Supplementary Materials

The following are available online at http://www.mdpi.com/1420-3049/21/9/1242/s1.
