# Supplementary material for: Screening of Potential Xanthine Oxidase Inhibitors in Gnaphalium hypoleucum DC. by Immobilized Metal Affinity Chromatography and Ultrafiltration-Ultra Performance Liquid Chromatography-Mass Spectrometry
Source: Molecules. 2016 Sep 17;21(9):1242. doi: 10.3390/molecules21091242 (PMC6273769; doi:10.3390/molecules21091242)
Supplement: Supplementary file 1 [file molecules-21-01242-s001.pdf]

Supplementary Materials: Screening of Potential Xanthine Oxidase Inhibitors by Immobilized Metal Affinity Chromatography and Ultrafiltration-Ultra Performance Liquid Chromatography-Mass Spectrometry in *Gnaphalium hypoleucum* DC.

Hong-Jian Zhang, Yi-Juan Hu, Pan Xu, Wei-Qing Liang, Jie Zhou, Pei-Gang Liu, Lin Cheng and Jin-Bao Pu

1. Metal-CTS-SiO_2 Elution Characterizations

The *G. hypoleucum* extract was dissolved in water and loaded on the IMAC column. Then the column was washed with pure water and 1% HCl-MeOH, and the eluent collected for analysis by HPLC (Varian Inc., Santa Clara, CA, USA). SiO_2 and CTS-SiO_2 without metal columns were used as control groups. The separation was carried out using a 250 × 4.6 mm YMC 5 μm C_{18} column. The column was kept at room temperature. The mobile phase consisted of 0.1% formic acid aqueous solution (A) and acetonitrile (B) using a gradient elution of 15%–60% B at 40 min. The flow rate was set at 1.00 mL/min. The detection wavelength was 250 nm.

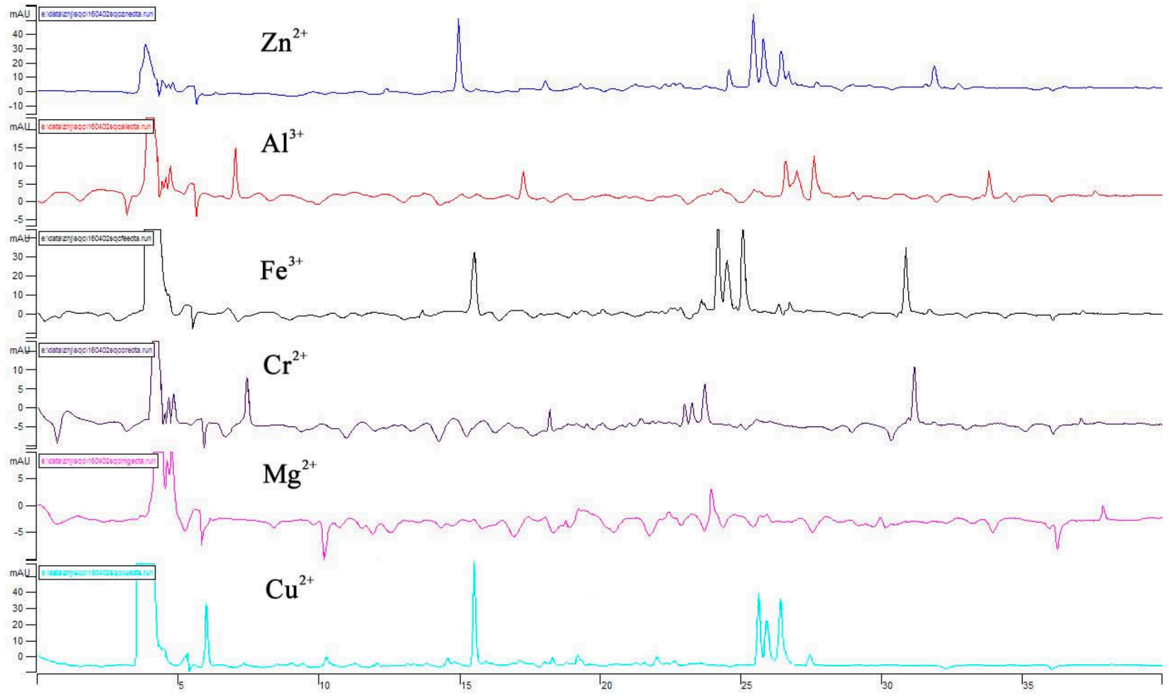

Figure S1. The HPLC chromatograms of 1% HCl-MeOH elution from an IMAC column packed with Metal-CTS-SiO_2 (Cu^{2+}/Mg^{2+}/Al^{3+}/Zn^{2+}/Cr^{2+}/Fe^{3+}) monitored at 250 nm.

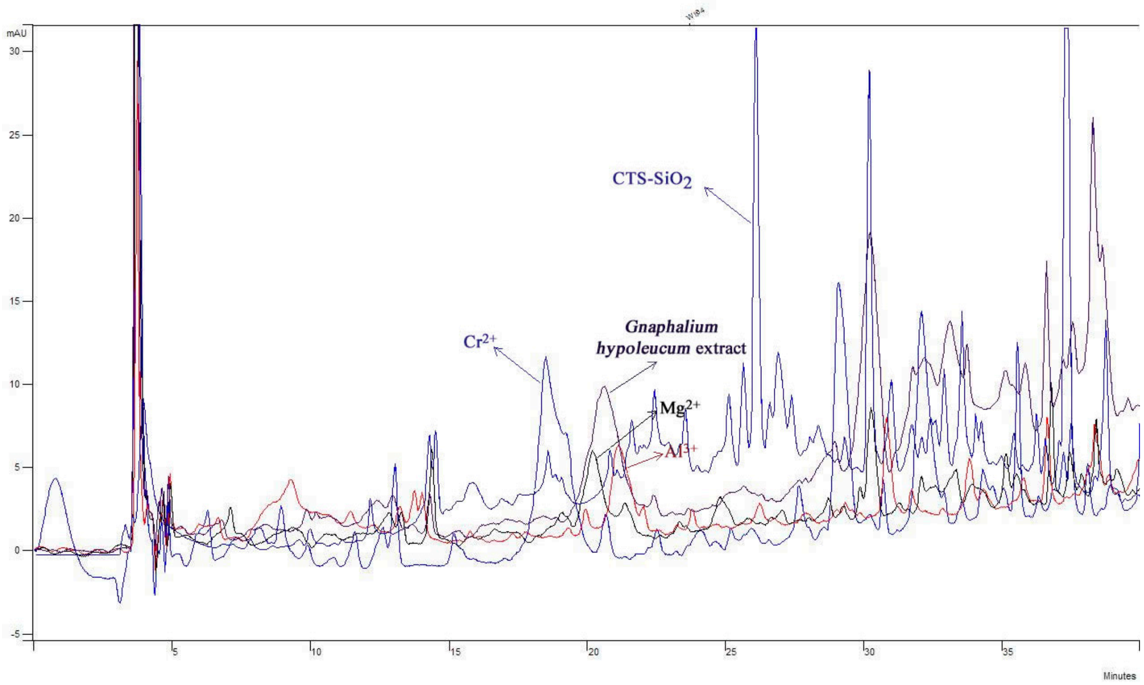

Figure S2. The HPLC chromatograms of water elution from an IMAC column packed with Metal-CTS-SiO_2 (Mg^{2+}/Al^{3+}/Cr^{2+}), CTS-SiO_2 without ion and *G. hypoleucum* extract monitored at 250 nm.

2. Determination of the Equilibrium Concentration of Metal Ions at

Samples were weighed directly into polypropylene flasks in aliquots containing approximately 250 mg for CTS-Zn and CTS without Zn ion. Aliquots of 3.0 mL of HNO_3 and 1.0 mL of H_2O_2 were added to each sample aliquot in the polypropylene flasks. The mixture was then placed on a heating plate for 3 h. The digested samples were transferred to a 50 mL volumetric flask with 0.5% HNO_3 and kept under refrigeration until use. The hollow cathode lamp for Zn was operated at 6 mA, with a spectral bandwidth of 0.5 nm. The selected wavelength was 213.9 nm for Zn. The standard solution was prepared at 100, 50, 25, 12.5, 6.25 μg/L using 0.5% HNO_3 to release. A blank submitted to the same procedure described above was measured in parallel to the samples and calibration solutions. The graphite furnace temperature program for each analyte is shown in Table S1. The Zn ion content was calculated using the following linear equation based on the calibration curve:

$$Y = 0.31X - 0.0026\ (R^2 = 0.9992)$$

where Y is the absorbance, X is the Zn ion content in μg/L. Samples were analysed in triplicate.

The concentration of the Zn ion in the resulting solution was 17.725 μg/g and under minimum linear range of CTS without Zn ion.

Table S1. Heating program of the graphite furnace.

Stage	Temerature/°C	Ramp/s	Hold/s	Ar Flow Rate/L·min^{-1}
1	85	20	5	0.3
2	95	0	40	0.3
3	120	0	10	0.3
4	300	0	5	0.3
5	300	0	1	0
6	300	0	2	0
7	1900	0	0.8	0
8	1900	0	2	0.3
9	1900	0	2	0.3

3. Determination of the Flavonoid Content of Extracts

G. hypoleucum extract (0.131 g), 1% HCl-MeOH (10 mL) and water (40 mL) were placed in a 50 mL volumetric flask and $NaNO_2$ (1 mL, 1:20) was added. Then $Al(NO_3)_3$ (1 mL, 1:10) was added 5 min later. After 6 min, 10 mL 1 mol/L NaOH was added and the total was made up to 50 mL with distilled water. The solution was mixed well again and the absorbance was measured against a blank at 507 nm with a Varian spectrophotometer. Since the luteolin content is as high as 30.23~181.40 μg/mL, it was used as the standard for a calibration curve. The flavonoid content was calculated using the following linear equation based on the calibration curve:

$$A = 0.0056C - 0.0384 \ (R^2 = 0.9992)$$

where A is the absorbance, C is the flavonoid content in μg/mL. Samples were analysed in triplicate.

The concentrations of total flavonoid in *G. hypoleucum* extract, CTS-Zn, Fe, Cu 1% HCl-MeOH and water eluent were shown in Table S2.

Table S2. The flavonoid content of samples.

Samples	Content (%)
G. hypoleucum extract	22.6
CTS-Zn 1% HCl-MeOH eluent	85.2
CTS-Fe 1% HCl-MeOH eluent	68.6
CTS-Cu 1% HCl-MeOH eluent	66.5
CTS-Zn water eluent	6.3
CTS-Fe water eluent	7.9
CTS-Cu water eluent	8.4

4. Rt, UV Spectra and *m*/*z* of Characteristic ions of the Reference Compounds Luteolin-4′-*O*-glucoside and Luteolin

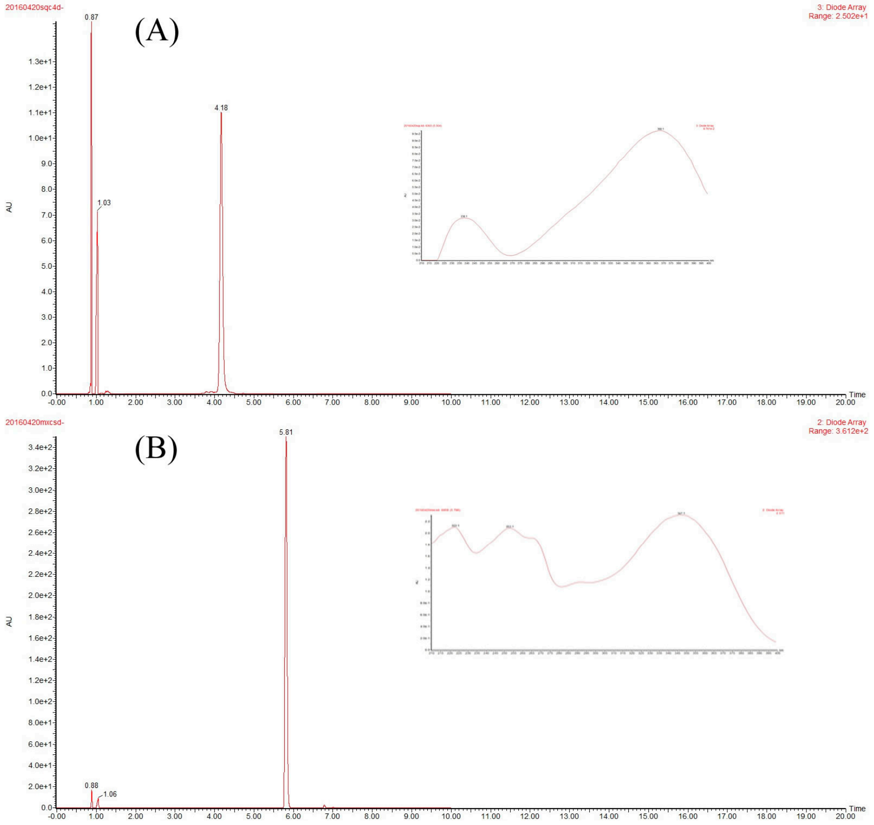

Figure S3. Retention times (Rt), UV of luteolin-4′-*O*-glucoside (**A**) and luteolin (**B**).

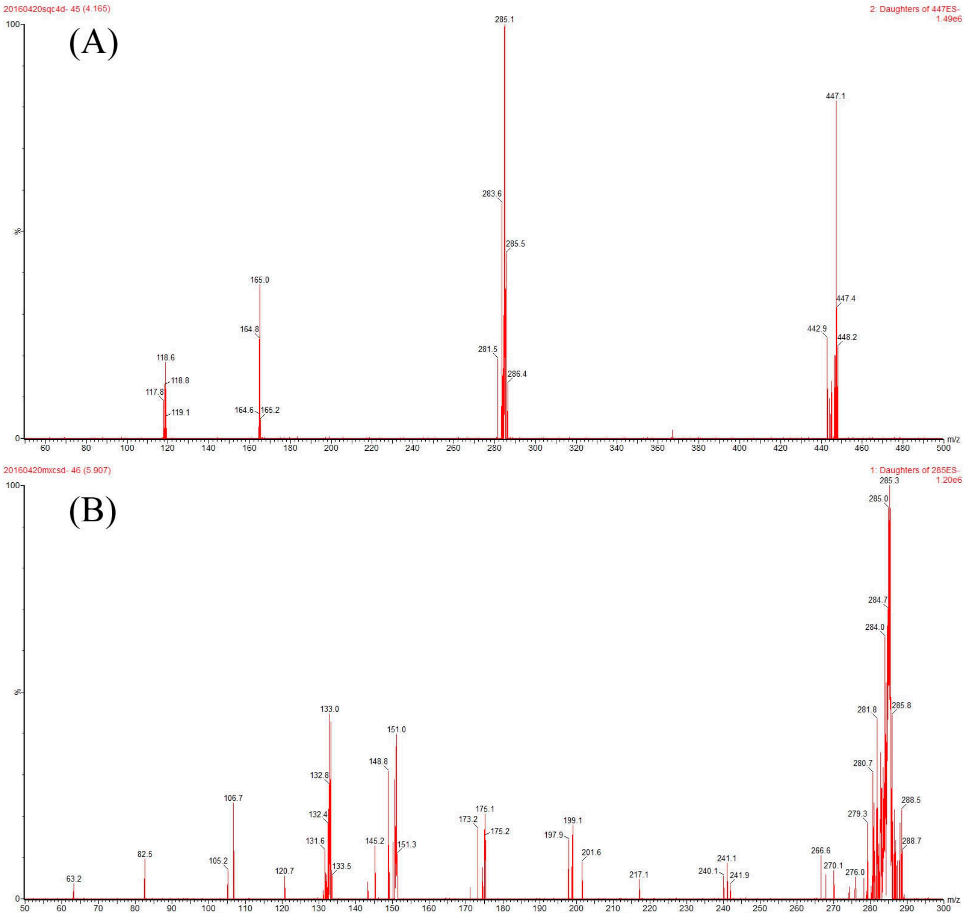

Figure S4. MS data of luteolin-4′-O-glucoside (**A**) and luteolin (**B**).

Table S3. Retention times (Rt), UV, MS data for reference compounds luteolin-4′-*O*-glucoside and luteolin by UPLC-MS.

Rt (min)	UV	MS	Elem Comp	Fragmentation Pathways	Identification
4.18	368.1	447.1	$C_{21}H_{19}O_{11}$	$[M-H]^-$	
	238.1	285.1	$C_{15}H_9O_6$	$[M-H-Glc]^-$	Luteolin-4′-*O*-glucoside
		165.0	$C_8H_5O_4$	$[M-H-Glc-C_7H_4O_2]^-$	
		118.6	$C_7H_4O_2$	$[M-H-Glc-C_8H_5O_4]^-$	
5.81	347.1	285.3	$C_{15}H_9O_6$	$[M-H]^-$	
	253.1	151.0	$C_7H_3O_4$	$[M-H-Glc-C_8H_6O_2]^-$	Luteolin
	233.1	133.0	$C_8H_5O_2$	$[M-H-C_7H_4O_4]^-$	
		106.7	$C_6H_3O_2$	$[M-H-C_9H_6O_4]^-$	
